# Socio-cultural and behavioural factors constraining latrine adoption in rural coastal Odisha: an exploratory qualitative study

**DOI:** 10.1186/s12889-015-2206-3

**Published:** 2015-09-10

**Authors:** Parimita Routray, Wolf-Peter Schmidt, Sophie Boisson, Thomas Clasen, Marion W. Jenkins

**Affiliations:** Environmental Health Group, Faculty of Infectious and Tropical Diseases, London School of Hygiene and Tropical Medicine, Keppel Street, London, WC1E 7HT UK; Department of Environmental Health, Rollins School of Public Health, Emory University, Atlanta, GA USA; Department of Civil and Environmental Engineering, University of California Davis, One Shields Ave., Davis, CA 95616 USA

## Abstract

**Background:**

Open defecation is widely practiced in India. To improve sanitation and promote better health, the Government of India (GOI) has instituted large scale sanitation programmes supporting construction of public and institutional toilets and extending financial subsidies for poor families in rural areas for building individual household latrines. Nevertheless, many household latrines in rural India, built with government subsidies and the facilitation and support of non-government organizations (NGO), remain unused. Literature on social, cultural and behavioural aspects that constrain latrine adoption and use in rural India is limited. This paper examines defecation patterns of different groups of people in rural areas of Odisha state in India to identify causes and determinants of latrine non-use, with a special focus on government-subsidized latrine owners, and shortcomings in household sanitation infrastructure built with government subsidies.

**Methods:**

An exploratory study using qualitative methods was conducted in rural communities in Odisha state. Methods used were focus group discussions (FGDs), and observations of latrines and interviews with their owners. FGDs were held with frontline NGO sanitation program staff, and with community members, separately by caste, gender, latrine type, and age group. Data were analysed using a thematic framework and approach.

**Results:**

Government subsidized latrines were mostly found unfinished. Many counted as complete per government standards for disbursement of financial subsidies to contracted NGOs were not accepted by their owners and termed as ‘incomplete’. These latrines lacked a roof, door, adequate walls and any provision for water supply in or near the cabin, whereas rural people had elaborate processes of cleansing with water post defecation, making presence of a nearby water source important. Habits, socialising, sanitation rituals and daily routines varying with caste, gender, marital status, age and lifestyle, also hindered the adoption of latrines. Interest in constructing latrines was observed among male heads for their female members especially a newlywed daughter-in-law, reflecting concerns for their privacy, security, and convenience. This paper elaborates on these different factors.

**Conclusions:**

Findings show that providing infrastructure does not ensure use when there are significant and culturally engrained behavioural barriers to using latrines. Future sanitation programmes in rural India need to focus on understanding and addressing these behavioural barriers.

## Background

In 2011 sanitation coverage globally was 64 %. While open defecation is declining across the globe, 15 % (one billion) of the global population still defecate outdoors [1]. While some countries have reduced open defecation to only a few percent, India and 26 other countries remain with more than a quarter of their populations practicing open defecation. Among the one billion defecating in the open globally, 66 % of them live in India. Nearly all (92 %) of these Indians live in rural areas [1].

Despite concerted government efforts for the last three decades to promote sanitation, India has barely managed to achieve its Millennium Development Goal sanitation target to halve the proportion of the world’s population without access to safe drinking water and basic sanitation by 2015.

Efforts to increase rural sanitation coverage in India largely started with the Central Rural Sanitation Programme (CRSP) in 1986. This subsidy-based supply-driven approach to promote sanitation did not yield sustained impact, and the CRSP was replaced in 1999 with the Total Sanitation Campaign (TSC) [2–4]. Along with subsidies to help households below poverty line (BPL) build latrines, the new programme was improved to make it participatory and community driven. Sensitising and mobilising through information, education and communication (IEC) was the major new feature. The results were not particularly satisfactory despite the new emphasis [2]. Over 11 years of the TSC execution, households with a toilet in rural areas increased from 21 % in 2001 to 40.4 % in 2012, however, data suggest that 20 % of rural toilets were not working [5]. In 2012, with further modification to the strategy, goals, and funding reallocation, the GOI renamed the TSC as Nirmal Bharat Abhiyan (NBA). Under the NBA, BPL households as well as families considered poor but without BPL cards are being targeted with higher government financial subsidies for installing a sanitation facility with the goal of 100 % sanitised villages in which no one open defecates [6].

Odisha, in eastern India, is among the lowest performing states in terms of latrine coverage [7]. In 2011, 85 % of rural households (HHs) defecated in the open and latrine coverage increased marginally by seven percentage points between 2001 and 2011, reaching 22 % [8]. Those that own a latrine often do not use it regularly [3]. Usage of latrines all over India is not encouraging. A national survey in 2010 found that even in villages designated open defecation free (ODF), up to 50 % of newly constructed latrines were not used [3]. In some high coverage villages in Odisha, 83 % of households had toilets, but only 48 % reported using them [9]. Similarly, an evaluation of the TSC in Odisha in one district (Puri) found that 37 % of members of households with latrines reported never using them and less than half of household members who reported using their latrine did so always [10].

It can be concluded that in India, adoption and use of latrines is low despite potential health, economic and social benefits of sanitation [11]. This study was undertaken to obtain a better understanding of the reasons for low latrine uptake and to identify and understand factors that motivate and constrain latrine use particularly with regard to government subsidized facilities in Odisha from the perspective of households themselves.

## Methods

### Study setting

The study was carried out across rural villages in the Indian state of Odisha between July 2011 and September 2012, mainly in Puri District. The study was approved by the Ethics Committee at the London School of Hygiene and Tropical Medicine and the Independent Ethics Committee at the Xavier Institute of Management, Bhubaneswar, India. The study adheres to the RATS qualitative research guidelines.

Odisha with a high tribal minority population (see below) has historically witnessed a higher incidence of poverty. It is among India’s states with consistently low achievement on both the HDI (human development index) and GDI (gender development index), and scored lowest in 2006 on the female-to-male ratio of power over economic resources [12]. Female literacy is also low relative to other states [13]. While Odisha has made gains faster than the average state in recent years, the disease burden remains high with infant mortality at 51/1000 births in 2013, maternal mortality at 303/10.000 in 2006, and above average prevalence of underweight children [12–14]. Odisha’s performance with respect to provision of safe drinking water has been satisfactory with 75 % of households having access to an improved drinking water source (*i.e.*, community taps and tubewells) in 2011 [13].

Puri is a coastal district close to Bhubaneswar, the state capital, and is famous for its Hindu religious and cultural heritage. The vast majority (84 %) of Puri’s population is rural. Agriculture is the major occupation and rice is the main crop. Most residents have lived in their village since birth, with the exception of married women who typically must leave their village to wed. Thus exposure to urban living is limited, however among men short-term migration mainly within the district or state for economic reasons is not uncommon [15]. Some residents commute weekly to one of Puri District’s four towns to serve in local government or for private employment and some own small enterprises. While improved water supply access is quite high (79 % in 2014) in Puri District with households using mainly public deep and private shallow tube-wells, or sometimes public taps supplied by government rural piped and treated water schemes for drinking water, a large segment of households continue to use open water bodies for personal and domestic hygiene [16] and sanitation coverage is lagging (estimated at 15 % in 2008, currently reported at 56 % as of March 2014 [17]). All study villages had some government infrastructure such as schools, pre-school nurseries (Anganwadi Centres), electric supply, improved deep tube-well or government piped water supply taps, and concrete road access, except for the one tribal study village located in Ganjam District, which had piped water supplies, high sanitation access, and a nursery, but few other government services. Tribal populations in India tend to live in geographic and economic isolation and have a distinctive culture, similar to ethnic minorities. This tribal village had benefited from a very successful integrated approach to water and sanitation development promoted by a long-standing and respected NGO in sanitation and was included for contrast.

In close proximity of most non-tribal villages are small towns and villagers (mainly men) visit the markets in these towns for daily needs. Visiting the state capital is uncommon even by men, with a visit to a large city perhaps at most once or twice in a lifetime. Married and adult women rarely move out of their village. Only in emergencies like medical treatment, institutional delivery, or to attend a wedding, would women travel out of their village. The case is different for adolescent girls. Those who study often leave the village for limited periods to attend college in a nearby town or more rarely, a city.

Villages are typically comprised of different castes. The caste feeling is said to be declining but casteism persists and social disparities continue in terms of improved water supply and sanitation access within and between villages, similar to other rural areas in India [18]. Higher and middle castes may stay together in the same hamlet, whereas low (scheduled) caste people always live in a different hamlet located at some distance from higher caste hamlets of the village.

Indian society is patriarchal and multiple generations of extended family traditionally live together in the same house under a male head and his spouse, including any married sons and their wives and children, along with unmarried son(s)/daughter(s) [19–21]. In Indian, the spouse of the male head of the household is commonly referred to as the ‘mother-in-law’ (assuming she has sons) while a spouse of her married son(s) is referred to as the ‘daughter-in-law’. When a son marries, his bride leaves her father’s home and village to live with her husband’s parents and siblings, and is typically under the command and control of her new mother-in-law and in-laws until she herself becomes the mother-in-law of the home [19, 21].

### Sample selection

Because we were interested in understanding barriers to use of existing latrines, Puri District villages with some latrine coverage were first identified and a subset selected as a convenience sample based on the dominant type of latrine facilities in the village:

- Type A : Self-financed latrines
- Type B : GOI subsidized latrines with improvements financed privately by the household
- Type C: GOI subsidized latrines constructed without further improvement

A local NGO with experience in the Total Sanitation Campaign (TSC) delivery was approached for a list of villages including ones where the TSC had been implemented between 3-5 years ago and information on the dominant types of latrine facilities and castes in each village. Villages on the list were visited to verify information regarding the dominant latrine type(s) and to identify a focal person to help recruit participants and liase for other field arrangements. Participants who owned one of the three types of latrine facilities (A, B, or C) were purposively selected and grouped by facility type.

### Focus group discussions

The purpose of the FGDs was to identify reasons for latrine use and non-use and low uptake of latrines, explore preferences for open defecation, understand different domains of latrine use, understand attitudes and cultural practices in the context of sanitation, and understand the role of ownership, design style, proximity of water and location of latrine structures, as they related to latrine use and non-use.

A discussion guide for the FGDs was developed for soliciting insights along the themes of latrine adoption and non-adoption and reasons behind it, including information needs, decision making, motivations and barriers for participation in subsidized latrine construction programs, latrine usage and usability, latrine improvements (operation, maintenance, and repairs), and reasons for preferring open defecation. FGD questions were first developed through a preliminary brainstorming session with a group of unmarried young women from villages in Puri. Their personal experiences of the sanitation situation in their villages and their own sanitation practices provided insights into sanitation behaviours and attitudes in rural areas and across different seasons which in turn informed topics and questions for the guide. Once the guide was developed, it was pretested with professionals and local practitioners with knowledge of rural realities and experience in participatory approaches to check the appropriateness of questions, the manner of questioning participants, and the flow of the discussion themes. The questions were simplified and sorted into main topics and sub-questions for probing to help participants further understand the focus. The guide was translated into Oriya (the local language) for the convenience of personnel taking notes during the FDGs which were facilitated by the first author (PR), a native Oriya speaker. PR also conducted the latrine observations and interviews (see below) with assistance from the last author (MJ) who observing most of the FDGs.

Twelve FGDs were carried out (see Table 1). Of these, one was held with front-line field personnel from four different NGOs implementing the TSC in the study area and another with women self-help group (SHG) members who had assisted the NGOs with implementation in their respective villages. The remaining 10 FGDs were conducted in five villages, separately with male and female participants. Village FGD participants overall varied in age, gender, latrine ownership, marital status and caste but were segregated into separate homogenous groups to facilitate open discussions.

**Table 1:** Overview of focus group discussions, participant characteristics and latrine ownership and type

Number	Focus group type	Latrine type owned	Gender	Number of participants	Village	FGD date
1	NGO field staff (4 NGOs)	-na-	Men	8	-na-	1 Jul 2011
2	SHG members (6 SHGs)	GOI subsidised, improved & not improved	Women	12	#1-5	2 Jul 2011
3	Married, high (Brahmin) caste	Self-financed	Women	9	#6	5 Jul 2011
4	Married, high (Brahmin) caste	Self-financed	Men	7	#6	5 Jul 2011
5	Newly married young, low (Scheduled) caste	GOI subsidised, not improved	Women	6	#7	3 Jul 2011
6	Married, Low (Scheduled) caste	GOI subsidised, not improved	Men	7	#7	3 Jul 2011
7	Married, general caste	GOI subsidised, Improved	Women	8	#8	6 Jul 2011
8	Married, general caste	GOI subsidised, improved	Men	8	#8	6 Jul 2011
9	Married, tribal	GOI subsidised, improved	Women	6	#9^a^	9 Jul 2011
10	Married, tribal	GOI subsidised, improved	Men	7	#9^a^	9 Jul 2011
11	Unmarried adolescent^b^, lower castes	none	Women	7	#10	29 Sep 2012
12	Unmarried adolescent^b^, mixed castes	none	Men	7	#10	29 Sep 2012
	Total			95	10	

^a^The sanitation programme in this village was implemented by Gram Vikas, a well-respected and long-standing NGO acclaimed for their contributions to the water and sanitation sector. They specialise in a unique and very successful integrated water and sanitation approach to promoting village-wide individual household latrines coupled with simultaneous delivery of a new piped water system comprising a yard, bathroom, and latrine tap for each household

^b^Ages were 17 to 21

Six to twelve participants were included in each FGD (see Table 1). Discussions were held separately with married adult men and women, and with unmarried young women and men in their own natural setting at a common and quiet place in the village. FGD times were decided based on participants’ convenience and availability. Government representatives in each village, such as the Accredited Social Health Activist and nursery workers, were consulted for recruitment of participants as per the latrine type criteria and caste. Seating was ‘U’ shaped or round so that participants, including the facilitator, could all see each other. Prior to the discussion, an information sheet containing the aim and objectives and other details of the study was read aloud, questions were answered or clarified as needed, and verbal consent to participate in the study was obtained from each participant as well consent to audio record the discussion. As all participants were above the age of 16, no parental consent was needed. At the end of each FGD, the facilitator (cum interpreter) and observer along with the note takers reviewed the discussion and descriptive notes of expressions or statements were prepared. Full audio recordings of each FGD were translated and transcribed verbatim into English for analysis.

### Field observation

Prior to and right after each FGD, several household visits were made to observe the functionality status, design, location and water access of GOI subsidized latrines as well as self-financed latrines and to interact with the owners to explore satisfaction, usage, and the design and situation behind constructing their latrine. From these observations, field notes were prepared for both village and home visits. Observation of each latrine’s condition and important conversation with latrine owners about reasons (or circumstance or situations) for installing latrines were noted as bullet points during each field visit. At the end of the day, detailed descriptive notes were prepared, and put together with the FGD transcript for inclusion in the analyses. The data from observations was used to get a comprehensive and complete picture of the issues, in particular those related to latrine design, construction, and performance, understand the social situations, and gain a different perspective of behaviour within a larger social and physical setting.

### Data analysis

For each FGD transcript, each idea (or statement) was highlighted and initially coded as a ‘motivation’ , a ‘constraint’ , or a ‘facilitator’ for latrine use or for open defecation and tagged, where relevant with the category of person (*i.e.*, age category, gender, marital status, caste, type of latrine, *etc.*) to whom it referred. Each highlighted text item and its assigned code was then transferred to a row in an Excel table to collect all highlighted FGD items. Working in Excel, items describing a similar idea within each main theme were then grouped and further coded manually and sorted to capture common sub-themes. For each emerging sub-theme, a summary explaining the behaviour, attitude, experience, context and ritual around observed defecation practices and patterns was prepared, providing the basis for the results presented in this paper.

## Results

### Open defecation practices in different seasons and times of the day

The majority of the study population defecated outside in the periphery of their villages in open fields or bushy areas to hide themselves and avoid being seen by others. Vacant areas around local surface water bodies were the most preferred defecation places, as water was readily available for anal cleansing and body bathing and clothes rinsing, key elements of local sanitation rituals especially for defecation in the morning. Women and men had separate open defecation sites which varied with the season, time of the day, and need of the individual. It was uncommon to find men and women using the same site for defecation, except in exceptional circumstances like floods, when there is a shortage of open space due to inundation, or health emergencies, when people are too ill to walk long distances or their bowel movement is beyond control, and they have to defecate urgently.

OD sites differed with the season. “The most difficult time for defecating outside (in fields) is the *chaturmasia* (Oriya for the rainy season or monsoons from July to September) as land is inundated due to excess water in low-lying areas.” (FGDs #2, 5, 11) In all FGDs this point was raised time and again by different participants. In such situations, they relied on raised land beside the road for defecation. Some stated that they defecated on a dried cow dung cake, and then threw it into the flood waters. “But after the floods are over and as water recedes, they resume defecating in fields.” (FGDs #2, 5, 6, 11, 12) During the rice growing season (September to early January), people reported not defecating in the fields and gave numerous explanations for avoiding them. First, grains are considered *laxmi* (goddess of wealth) and participants strongly believed defecating in crop fields was a ‘sin’ when standing crops were still there. Fear of snakes or insects was another reason for avoiding defecating in rice fields. They also found it inconvenient to squat in the midst of the rice plants. Also, owners did not allow anyone to defecate in their fields because if a person who harvests knows that someone has defecated there he will feel disgusted with the faeces around him while harvesting. In contrast, after the harvest, in the period that follows (January to March), people reported feeling very comfortable defecating in harvested fields since the crop has been removed and the breeze makes it pleasant. In winter season (October to February), as the nights are longer, and people rise comparatively late, they preferred defecating somewhere closer to their habitation. With the cold morning atmosphere there was an unwillingness to walk long distances for defecating.

Not every household has a private well or tube-well on their property and many villagers rely on local surface water bodies (typically ponds, irrigation canals and rivers) for hygiene activities like bathing, washing clothes and utensils, and even anal cleansing post-defecation. Older mothers-in-law often combined all the hygiene activities to be conducted outside home with going for OD. They would leave clothes and utensils to be washed near the pond, and go to defecate in nearby fields. After defecating, they would cleanse themselves in the pond and then finish their activities in the same place.

Similarly, OD sites changed with the availability of water in local water bodies, *i.e.* during the dry season (early March to June). Rural Indians require water for anal cleansing and post-defecation body washing and clothes rinsing, so when larger, flowing water bodies like irrigation canals and rivers dry up in the study area, villagers rely on ponds located nearer to the village, while those who feel shy and want to avoid being seen, walk long distances where they are invisible to others and water is available. During the late dry season, after the release of water into irrigation canals in the region, preferred sites for OD become the canal embankments. Table 2 summarises the seasonal variations in OD sites.

**Table 2:** Overview of open defection (OD) practices by different age, gender and occupational groups

Age	Gender	Occupation	Defecation places	Preferred time of day, alone or group	Preferred OD sites
0-5 years	Both	NA	At home on ground or floor	None	Inside home, vacant places next door, road sides or village streets
5-16 years	Both	School students	Field, bush	None	Vacant fields preferably closer to house
17-20 years^a^	Girls	School/ college students	Field, bush	In group, preferably in evening hours	At sites close to house
17-20 years^a^	Girls	Non- students	Field, bush	In group	Go far from the village during the post-harvest and summer season
17-20 years^a^	Boys	School / college students	Field, bush	No preferred time, alone or in small group	River beds and canal embankments
Adult	Men	Farming	Field, bush	Morning, mostly alone	Canal or river embankments; open fields
Adult	Men	Non-farming	Field, bush	Morning, mostly alone	Road sides, canal embankments; fields next to water bodies
Adult	Women	Housewives	Field, bush	Mostly alone in the morning, but in groups in the evening	Bamboo bush or bushy areas
Adult	Women	Newly married daughter-in-law	Field, bush	Accompanied by female family member (chaperone) very early (4-5 am) before dawn; in small group with family chaperone in evening (5-6 pm)	Field closer to house in early morning; far from house in evening
Adult	Men and women	Elderly, disable or sick members	Field, or in house (on bed, cloth, paper)	Health condition determines the location of the OD site	Close to house; in backyard

^a^Referred to as “adolescents” in main text

Women preferred defecating in a safe and convenient place where they could hide themselves from the sight of males as they did not like to be seen by others during the act. For this reason they did not mind walking long distances to reach fields away from their habitations to ensure that no one could recognise them. While defecating they hid themselves behind a bush or the cover of a tree. If someone passed by, they had to stand up even in the middle of the act until the person left. For men who were farmers, defecation sites were unused land somewhere close to their agricultural fields. Most farmers leave home early in the morning to work in their fields and defecate on their way to their lands. Many women had the habit of defecating twice a day, in the morning and in the evening. Women’s preferred early morning when it is still dark and at sunset before it is dark, to ensure they were less visible to others under the cover of darkness. Evening defecation is done as a precaution by many to avoid having to go in the night and cause inconvenience to other family members who would have to accompany them in the night to OD.

#### Routines of rural women

An overview of open defecation practices by different age groups is presented in Table 2. The general consensus among female FGD participants on the defecation practices of other females in their village is illustrated by the comment: “Going for defecation in the evening is the most awaited time by women. Women go in groups (mostly of 4–5) and in pairs (sometimes) to defecate in fields to chat with their friends/relatives about the ups and downs of their daily lives and to feel free from household chores.” (FGD #5) These informal groups of women form on the basis of marital status in the family (position/hierarchy), bonding with other family members in the house or with relatives, and eventually, friendships with other women of a similar age in their hamlet. A newly married daughter-in-law would not be able to join a group immediately after her wedding, but as time passed (sometimes several years), she would establish rapport and join a pre-existing group. Similarly, unmarried college or school girls went to defecate in groups of their own age or might accompany a newly married young sister-in-law. A newly married daughter-in-law could never go out to defecate without a female family member as there are restrictions on their movements and on leaving the house alone, being new to the place and for safety reasons; even being seen by men in the village is deemed problematic for her. To avoid any chance of being seen by other villagers while going for open defecation, newly married daughters-in-law had a different and very early time for defecation (*i.e.* 4:00 am). Very young girls did not have a separate group but accompanied their mothers, aunts and grandmothers.

For safety and privacy reasons, going in groups for defecation was preferred by most women as they felt protected from the fear of theft and being harmed or attacked by mischievous men, and they felt less likely to be traced or seen by anyone while in a group. In other instances, especially early morning and after dark in the night, if an adolescent or young married woman needed to go for defecation, someone from the family was required to accompany her to the OD site to safeguard her as these members are considered the most vulnerable to such female attacks and threats. The mother-in-law or sister-in-law usually accompanied the new daughter-in-law to the OD site, but another family member could accompany an unmarried adolescent female member. The following quotes illustrate the kinds of threatening situations that women, especially younger ones, faced when going for OD in rural villages, involving personal theft: “While the newlywed daughter-in-law went to the field to defecate in the evening, someone hiding in the bushes arrived suddenly and snatched away her gold necklace. The girl could not identify him and nobody found the thief.” (FGD #7) One incident of sexual harassment was reported where a participant mentioned: “When my neighbour was defecating, a mischievous man came and held her hand, and misbehaved. She was alone and there was no one to rescue her. She went and complained to her husband. But they could not fight back.” (FGD #11)

Participants expressed no problems for women with OD at night, since they could go close to the house which was often more convenient than using the latrine (for those who had one) at night as they could hide themselves in the darkness even close by the house.

Most women with subsidized latrines indicated they preferred going out for OD in the evening hours as they had comparatively less chores than in the morning. They would finish household chores in advance and set a time for departing to the OD fields with their women friends or relatives even if they did not need to defecate themselves. They said they used this time to chat with others and disconnect from household chores, relax, and socialise. Some said they used this time to share and release their stress from family problems and for venting out. For many women, especially a daughter-in-law, this could be their only chance to escape the confines of the house and the scrutiny and control of their mother-in-law.

#### Routines of rural men

Defecation outside is a common practice among most rural adult men. Being farmers, they need to visit their crop fields early in the morning. They generally do not go for OD in groups as women and girls do. They also do not wait for someone to accompany them or wait for the cover of darkness. Morning is the most preferred time for adult men. Unlike women and girls, they do not “schedule” defecation but rather defecate whenever the need arises, either on their way to or returning from their fields. Many are habituated to brush *gudakhu* (tobacco paste) on their teeth, smoke *bidi* (cigars), or drink tea before going for defecation.

Men who have a job or work outside the village prefer defecating at a site somewhere close to their houses so that they spend less time. The most preferred sites for these men are the sides of a nearby road, canal, or river embankment where open water bodies are available nearby for anal cleansing.

Practices among adolescent boys and unmarried men are different from those of adult men. On their way to the OD site, if they meet some other (male) friend, they invite them to join for a chat. If they go in a group, the size is small compared to that of women. The most preferred sites for these boys are the river beds, or canal embankments, as these places have fewer people and have water available for post defecation cleansing and bathing rituals.

#### Routines of young children

Infants and very young children (toddlers) are made to defecate inside the house or compound on a paper or cloth, or directly on the ground, depending on the extent of their mobility. Their faeces are usually disposed either in the waste/garbage pit, or a vacant plot next to the house. When the faeces is watery and cannot be separated from the cloth, the same is rinsed and then washed in water bodies.

Mothers train the child to defecate at an early age, by being made to sit on the mother’s feet and squat. Later as they become older, they are taught to squat on bricks instead of the feet. A few mothers used a potty (a plastic portable squatting pot, designed especially for children) and the stool collected was disposed of in a vacant site close or next to the house.

#### Routines of old, disabled and sick persons

Unlike younger people, old people defecated closer to their houses. They did not feel ashamed of being seen and they did not have a fixed schedule. Members with some kind of disability or elderly family members who are unable to walk on their own are made to defecate on a paper or cloth. Health condition determines the location of the OD site (usually in the backyard) and its distance from the house. Rules are relaxed for family members as to where they can defecate during critical times. Social norms for acceptable and unacceptable places are flexible for sick family members and they are permitted to defecate inside the house. The faeces are then disposed of in the garbage pit.

#### Sanitation rituals and practices of higher and lower caste people

Defecation practices in rural areas follow elaborate rituals. They often involved symbolic acts of purifying the body and clothes with water following defecation or contact with human faeces or even simply with the latrine itself (such as entering to clean or dispose of a young child’s faeces), especially among the higher castes. In a physical sense, however, these may not necessarily result in real cleaning. Similarly, changing clothes is one of the most important parts of most defecation routines of both men and women among higher castes, but members of lower castes do not also follow this ritual as rigorously as higher castes. An overview of sanitation rituals by caste is presented in Table 3.

**Table 3:** Sanitation rituals among different castes

Caste	Men	Women	Children
Brahmins (the highest caste) and, other general castes (with better economic status)	Change of clothes pre- and post-defecation, and body cleansing with water after defecation is an important aspect of the defecation ritual practiced by rural people. The common belief is that clothes worn while defecating become impure, and by rinsing or washing with water, they are ‘purified’. Therefore, they have a separate cloth (a *dhoti* or *lungi*, meaning towel) to be worn while going for defecation. This cloth is usually kept outside the living area, away from the main house and away from the reach of children and adults, so that no one touches it. In case of urgency, where they fail to change their clothes, the clothes have to be rinsed with water after defecation. Wearing the same clothes without rinsing or washing is forbidden and they are restricted from entering the house. Full body bath is not necessary. The sacred thread (called *paita* ^a^) is wrapped around the right ear twice while going to defecate and once when they urinate. After anal cleansing, followed by full body washing with water, the thread is taken off the ear and made wet and put back on the shoulder again. They are restricted from touching the water point after defecation (see details in next column on women’s rituals).	As described for Brahmin men, body cleansing with water after defecation, is strictly practiced among females as well. Females of all age groups (excluding the very young) have to change their clothes, each time they go to defecate. Adhering to this ritual, the common practice is to keep aside an old unused gown, saree or dress, and change into it for defecation. For those with latrines, stepping over the squatting pan is considered *chuan* (*i.e.* getting impure) and both the body and clothes worn get impure. They are forbidden from entering the house wearing impure clothes. They can purify only by rinsing the dress/clothes they have worn or by changing them. For this reason, they prefer urinating outside the latrine mostly in the backyard and the latrine is used only to defecate. Similarly, they prefer to dispose of young children’s faeces which are not considered impure, outside of the latrine, to avoid having to perform these lengthy rituals. They are restricted from touching or accessing water points (tube wells, or wells) at home with clothes worn while impure from defecation. Therefore, they have to collect and store enough water for not only anal cleansing and flushing, but also for bathing and washing their clothes before going for defecating when using the latrine. In case they did not fetch enough, someone else has to assist them to fetch the water they need to use the latrine.	Changing of dress or clothes is not mandatory for infants or young children. Children who can defecate on their own have to remove all garments when they need to defecate. Faeces of children above 3 years are considered impure as by that age, the child starts eating rice and the faeces smell. Mothers develop a disgusting feeling for it. For a baby who defecates on the ground or floor, the mother may pick up the faeces with straw or other old materials and dispose of it in the bush or the waste/garbage pile. Mothers are unaware of the need for safe disposal, or of methods to do so, and prefer to avoid changing their own clothes which would be necessary if they entered the latrine to dispose of children’s faeces or help young children use the latrine. It is more convenient for them to throw these faeces on vacant land next to the house or in the backyard, and have young children defecate outside.
Other castes (poor)	Changing of clothes is a common practice, but many poor families do not have extra dresses for changing during defecation. So, they use the same clothes each time they go for defecation and wash their fully clothed body (both body and clothes together) with water.	Women do the same as men.	They don’t strictly follow the rituals of changing clothes, each time they defecate. Mothers are not very strict or particular about the changing of clothes of children.
Lowest castes (Scheduled ^b^) (poorest)	They mostly are the landless and work as labourers or share croppers. They usually defecate on their way to the fields and bathe before returning home. They don’t have any strict practices of changing clothes. Those who are more hygiene conscious prefer to change their clothes.	Women also work as agricultural labourers, and their defecation practices are similar to those of men.	Children accompany their parents to the fields, and their practices are the same as their parents.

^a^ *Paita* is a thin consecrated cord composed of distinct cotton strands and worn by adolescent and adult male Brahmins. The thread is worn across the torso and over one shoulder, after the thread ceremony conducted when a boy is seven years old, but this is changing with time

^b^ Scheduled castes are also referred to as “dalits” or “harijan”

Adherence to and practice of purification rituals and rules which are time and/or water consuming, as indicated in Table 3, discouraged use of the latrine for urination, by child, for faeces disposal and at night. Defecation times and rituals of changing clothes among higher and lower caste members remain almost the same. However practices vary across individuals and their type of occupation as seen in Table 2. As habitations in villages are clustered according to caste, and the hamlet of the lower castes (*i.e.* scheduled castes) is situated at some distance, defecation sites also differ. It is very rare to find people of lower castes going for defecation together with higher castes and using the same sites.

### Open defection due to no latrine

Lack of access to a latrine was stated as the primary reason why people who did not have a latrine practiced open defecation (OD) by participants, and lack of cash income on the part of economically poor families was the most stated reason for not opting to install a GOI subsidized latrine, despite the GOI subsidy (valued at Rs. 2,200 or 3,200 at the time the TSC program had been implemented in study villages) since participation in the TSC requires making a small contribution to toilet construction. Others thought sanitation costs were high and unaffordable.

### Reasons for maintaining open defecation despite owning a latrine

Rural people had their justifications for practicing open defecation despite owning a latrine, especially those with a GOI subsidized latrine. One important reason related to gaps in the government TSC sanitation intervention delivered to them. Many did not use their subsidized latrine because they complained that the structures were not built properly, that they lacked a roof, a door, and any walls sometimes, or the pits were too small. Our observations confirmed these complaints regarding inadequate design and incomplete construction of subsidized latrines, and sometimes also deficiencies in the quality of construction, for example, pans were installed at or inches from ground level resulting in an insufficient slope between the pan ‘S’ outlet and off-set pit inlet. Participants also complained about the small design of the cubicle which made squatting difficult and uncomfortable and, where the latrine was unfinished and lacked a door or sufficient height walls (a frequent occurrence we observed), that visual privacy was not ensured. Women had a distinct need for visual privacy, in contrast to little or no need for men. Due to the shallow depth of many of the subsidized single pit latrine designs (often three rings, each 25 cm height, for total depth of 75 cm), some feared that if all members used the latrine all of the time, the small pit would quickly get filled. So, men preferred to defecate outside in the dry season, leaving the latrine for women to use. In one case of a scheduled caste family who had never seen or used a latrine before, no instructions on how to use their GOI subsidized latrine (a pour-flush on-set model) were provided by the NGO who installed it, and so they broke the pan’s water-seal because they could not understand how else to make the faeces go from the pan into the pit below.

In the study communities, people are washers (*i.e.*, using water for anal cleansing and post defecation ritual bathing) so that the absence of a water supply in or next to the toilet was another major reason for non-use of GOI subsidized latrines. Households we interviewed reported that their subsidized toilets required fetching about 12 L (one bucket) for anal cleansing and flushing the waste from the pan, and another 12 L (2nd bucket) for post-defecation washing of their body and clothes. This water had to be fetched or available at the latrine before entering to defecate, since a person who has defecated was contaminated (polluted) and therefore could not touch the water supply point without first ritually purifying themselves by bathing or changing clothes. Further, many poorer households only possessed one bucket, and a bucket, once carried into the latrine was considered contaminated and could no longer be used for other tasks.

Although in most villages there were multiple public water supply points (*i.e.* public tube-wells and government piped water taps) and points were located from 50–300 meters of most habitations (with exception of distant hamlets of the scheduled caste), we did not observe any habit of transporting and storing water at home for personal and domestic hygiene activities. Rather these activities were done at the public water point or in local surface water bodies for households that did not have a privately installed water supply at home, and only very small volumes of water were stored at home for the purpose of drinking only. Thus, water fetching for latrine use is perceived as an additional time consuming new task for them, whereas in going for open defecation they are spared from this workload, because OD sites are selected near open water bodies where they can easily and conveniently perform anal cleansing and bathing before returning.

For some, their occupation was a hindrance to latrine use which did not suit their daily routine. For farmers, who leave the house first thing in the morning for the fields, using the latrine was inconvenient and extra work and time. They did not feel the need to come back from the farm, only to defecate in the latrine and have to fetch water. Other than these reasons, people were not able to give up their old habits of OD. This seemed to be especially true for older members of the household. Some participants remarked: “They (elderly in the family) will continue going out (*i.e.*, not using the latrine). Motivation to use may arise among other groups of people, but to bring in a change in old is extremely difficult.” (FGD #3)

Socialising was another important factor contributing to low latrine use, especially among the female population who remained confined to the four walls of the house. OD especially in the evening was a rare opportunity for them to leave their houses at least for some time and be free from household chores and responsibilities, and mix with others, as mentioned above. Reasons for people with latrines to defecate outside in open fields are detailed in Table 4.

**Table 4:** Reasons for members of households with GOI subsidized latrines to continue open defecation (OD)

Topic	Men	Women
Socialising	After the day’s work in the field, some men go to defecate in the fields with (a few) other friends. The male group size is small, 3-4 people at the most, comprising very close friends. Men use the moment for exchanging news and smoking cigarettes. For men, unlike women, going in groups is not a regular practice.	Females go for OD in groups, especially in the afternoon/evening time. Group size for OD varies between 6-10 women. Defecation in the open, in groups, twice a day is common, but OD in the evening hours is mostly used for socialising, sharing information and stress release, and they like to take more time at this time of day. Even though some women will not defecate at this time, they still accompany others to the defecation sites, which tend to be farther away from the village. For daughters-in-law, evening OD can be a rare opportunity for them to leave the house and the control/command of their in-laws, and relax from chores.
Purity and health	Containing faeces in the latrine pit inside the compound is perceived to be ‘impure’ and considered to be ‘disrespectful’ for the worship shrine at home. People feel latrine pits are the breeding grounds for mosquitoes^a^. With open defecation, they believe faeces (impurities) are left outside, away from homes and mosquitoes can’t breed.	Perceptions regarding impurity are similar to those of men. During the day, women are often confined to the home and remain engaged in chores. They feel by going for open defecation, especially when they have more time in the evening, they can get fresh air and exercise, as this is the only time when women can walk for some distance. Thus, OD is seen as good for health, and walking long distances for defecation is not necessarily regarded as an inconvenience.
Convenience/extra work	Men (adults and aged members, mostly the head of the household) are accustomed to going to farms or agricultural land immediately in the morning, after they are awake. All body cleaning activities like defecation and brushing teeth are done outside the house/property. On the way back from agricultural fields, they bathe and wash their clothes and return to the house for food in the afternoon. On account of these factors, using latrines for defecation in the morning does not suit their daily routines. Men are often concerned that the small pit (of subsidised latrines) will fill quickly if used by all family members regularly. They therefore preferred defecating in fields and letting women use the toilet. Emptying the pit was considered by some a constraint for latrine use, as only people belonging to lower caste groups can be engaged to do it.	Fetching water for family members for latrine flushing is difficult. Often, the typical source used for personal hygiene is different from that used for drinking water, and is a local surface water body. Drinking water sources like a public tube well or public tap are located in public areas in each village, but these can be at some distance from many houses, making daily transport and storage of sufficient amounts of water necessary to be able to use the latrine at home, unless the household has installed their own private tube-well inside their compound. Daily transport and storage of non-drinking water at home for non-drinking domestic needs, such as bathing or latrine usage, was never observed in any study communities and reported not to be a local practice. Making or helping a child use the latrine and then having to flush it, is considered more time consuming for mothers as it requires extra effort including her own purification after entering the latrine. Therefore, they find it more convenient to have the child defecate in the back yard and throw the child’s faeces into a garbage heap, than to have them use or dispose their faeces in the latrine. Women felt it is more convenient if children defecated on the road side or in fields, and then cleanse themselves in the public pond or another open water body in and around the village. Traditionally, cleaning and maintaining hygiene in the household is a responsibility assigned to women (predominantly). Thus keeping toilets clean is also considered a women’s job, and is seen as adding to their existing household chores.
Structural and design problems (small toilet size, no roof, water availability, *etc.*)	Overall, the construction of government subsidised latrines was of poor quality^b^, and in many cases it was not complete. The latrine design intervention delivered was a pour flush latrine cubicle with a single on-set or off-set pit with three cement rings (each 25 cm height), but without a roof or water facility. The covers of latrine pits were of such bad quality that they were quickly damaged. In some case, the door and even the walls of the toilet were missing. For these reasons, both men and women abandoned the toilets. Those with a functional toilet but without a roof, lived with an expectation of receiving funds from the government someday for the roof, and so postponed using the latrine until its construction was completed. Some feared the NGO-sponsored masons when they returned to finish the structure, would not install the roof of a ‘used’ latrine. This prevented some from using the latrine until it was complete.	Women prefer using latrines only if they are fully complete, *i.e.* have a roof, a water point nearby and a door that can be locked from inside. When children start consuming foods (such as rice) beside the mother’s milk, the child’s faeces tends to have an unpleasant odour. Mothers have discomfort in handling faeces and it is at this time they begin training their children. Additionally, mothers do not find toilet designs to be safe for young children to use on their own, and they delay training the child to use a latrine until they are about 5 years old. The latrine cubicle is too small for squatting or even keeping a water bucket. Unlike in open fields, they cannot move freely inside the latrine and so do not like using it. Water bodies are typically located close to open defecation sites, and so OD does not require fetching any water. Fetching water for toilet use is seen as a time consuming new chore. This hinders their use.
Privacy	Men have a lower need for privacy than women. Defecating and urinating in the open even during the day is not shameful. Sometimes latrines are located near the house entrance, and anybody passing by can see members entering or leaving the latrine.	Incomplete latrine structures do not provide sufficient privacy for women. Also, if the latrine is located within the compound it may be perceived as inappropriate to be seen by men while entering and leaving the latrine. Going to defecation sites is preferred, as men cannot see them there.
Habits	Rural men have the habit of going for OD. Changing habits is very difficult especially among elderly men. Men have different habits prior to actual defecation or during it which are not suitable to using a latrine. These include smoking *bidis* (cigarettes) which they find doing in the latrine difficult, unlike at open defecation sites.	Rural women especially the elderly are addicted to brushing *guddakhu* (tobacco paste) on their teeth before defecating. They believe *guddakhu* facilitates the bowel movement, which they feel freer and more comfortable doing in the open air or open field.

^a^We saw several subsidized pour flush household latrines in study villages, mostly on-set models, which were swarming with very small insects which may have been mosquitoes or flies

^b^Poor quality construction which we observed included use of insufficient cement for constructing the walls and the pan platform floor, rings supplied by the NGO for the pit were of bad quality (poor cement), so that some were already broken at the time of delivery but still used, and in some households, because the pit had not been dug when the NGO arrived with the mason to install the subsidized latrine, the rings were left stacked on the ground

### Reasons for choosing to use a latrine

However, some households perceive latrines as beneficial and have adopted them. Working and living in hostels in towns and small cities or in government quarters with latrine facilities often gave them exposure to latrines. As open defecation is difficult in towns, they were initially compelled to use the facilities, but then became habituated to using latrines and came to prefer the practice. Upon return to their village after retirement, they were among the first to invest in and build latrines on their own. Other factors we found that motivate households to install and use latrines are presented next.

#### Convenience and privacy

A latrine facility in the house (or compound) was more convenient and saved time in walking long distances in search of a proper and clean site to defecate, especially in the monsoon season when the area is waterlogged. There is a shortage of open space during monsoon and also during rice growing seasons, and squatting in knee-deep water or in the rice fields is difficult. Most female SHG participants (FGD #2) and men and women from Brahmin households (FGDs #3, 4) who had a toilet were using it at the time of the study, but at some point in the past they had practiced open defection. Participants with latrines from other FGDs, both men and some women, were not regular users of their latrine, using it mainly only during the rainy season, compelled by the lack of open space due to land inundation and rice cultivation (see above) which for women, especially, limited the number of alternative private places to use. They preferred going outside at other times of the year.

Interest in having a latrine was observed among people with some kind of disability. Due to difficulty walking long distances, they preferred using a latrine. For ailing family members, with diseases like arthritis, or leg fractures, mobility was a major issue and presence of latrines eased their daily lives. This was not, however, the general case for elder members who were not impaired and continent to open defecate nearby, and disinclined to changing their habits (see above).

Adolescent girls and adult women found latrines useful during menstruation, especially to clean their menstrual rags, since there was always movement of people at ponds and public water supply points which would make them ashamed to be seen cleaning them. The latrine’s proximity to the house and availability of a water supply point at or next to the house were thus key reasons that attracted some people to use their latrine. Visual privacy for women also seemed to be a fundamental reason in favour of using latrines, especially during the rainy season when there is a lack of private places to defecate. Household heads with a new daughter-in-law also did not like her to be seen or traced by other men in the village while defecating in the open, and thus saw value in having a latrine for her. Latrines, when well designed, could also be more peaceful than open fields as women did not have to stand up each time someone passed by. With a good latrine and water available at home, they were also able to defecate on their own (*i.e.*, without waiting for a female family member to accompany them to an OD site) and whenever they wanted, even at night. One poor household in a village, despite their poverty condition, build a latrine because they felt that if they had a latrine, they would be able to defecate peacefully. (FGD #1)

#### Dignity and status

Male heads usually are in charge of safeguarding the privacy and safety of their daughters-in-law, so they are often the instigators who feel the need to build a latrine for the women in the household, particularly for the protection of their newlywed daughter-in-law, rather than women themselves demanding it. Some toilets were found to be built just before a wedding, specifically intended for use just by the new daughter-in-law. In other instances, for better off people who had developed the habit of using latrines while in urban areas and built their latrine upon returning to the village, a more modern status and dignity for both female and males were the factors behind toilet installation. In these cases, both men and women regularly used the latrine. The influence of women on other women in one’s social network to build a latrine was also observed. If a few women members of one SHG obtained a latrine, this would impact other members in the group to want a latrine. Not wanting to fall behind status-wise with others in their social group, they would persuade their husbands to build a latrine.

#### Disgust

People expressed a feeling of disgust with the sight of faeces all around the OD site especially in the rainy season. Thought of a fly sitting on faeces and then on food, and of water in open fields contaminated with the faeces of different people drove some to build a latrine.

#### Exposure and awareness

Latrines were adopted among the population who had some kind of exposure to them and understood well their advantages. For example, females who had become accustomed to using a latrine at their parents’ home preferred to have a facility to use at their in-laws place which became their new home upon marriage. Others were exposed to latrines in urban areas during their formal education when they stayed in hostels, or when they visited the home of a relative who owned a latrine. There was also a segment of more educated people who were sensitised by NGOs about the disadvantages of open defecation related to health and disease transmission, and how OD was shameful for female family members, and became convinced. Situations of exposure and awareness from outside the village of the advantages of latrines were expressed predominantly among participants with SF latrines (FGDs #3 & 4).

#### Health and hookworm

Health and hookworm as a reason to build a toilet came up very rarely. Rather there were arguments by people saying that they had been defecating outside for ages and never had any health problem. There was only one case of a household installing and using a latrine for health reasons. Their adolescent son became very sick from hookworm and the doctor told them that to avoid hookworm they needed to stop OD and build and use a latrine.

#### Safety and protection

Even though participants reported it was very rare that women were harassed or faced a threat to their safety across the study villages when going for OD, fear of being attacked by someone in the dark persisted. A few male heads, out of concern for the safety and protecting the honour of female family members, especially a new daughter-in-law, built latrines, but these were designed with an attached bathroom so that women could bath and wash their clothes in fully privacy at home immediately after defecating in the latrine. There was also fear of insects, snake bites, leaches and other pests during the waterlogged season, and difficulty wading through standing water to find a place to defecate, expressed mainly by women. In these cases, setting up a latrine at home was deemed a better alternative. With a latrine, there was also no need for someone to safeguard or accompany the female member when going for defecation.

#### Routines

For a daughter-in-law in some households with a latrine, using the latrine in the morning was very convenient for her morning routine (but was not desirable for use in the evening) given her very busy morning schedule and sole responsibilities for cooking for the whole household which required that she finish defecating and bathing before she could begin to cook. With a latrine, she did not have to wake up as early to go out and finish OD and bathing in public before the light of day, saving her time and allowing her to sleep in a bit longer. For those members who commuted to work or college in towns, and also needed to save time in the morning, using a latrine made more sense as long as a water source was available in or close-by the latrine.

### Facilitators of consistent latrine use

In addition to the above cited motivations for latrine use, there were other facilitators that encouraged toilet adoption and use. Latrine design, location of the latrine structure close to the main house, and availability of a water source at the house or in the latrine were positive factors for adoption and consistent year-round use.

Most SF and many improved GOI subsidized latrines we visited had some signs of use, such as a broom, slippers, soap, a small water container, or a toilet cleaning agent. Most SF latrines we observed, although also pour-flush technology, also had a very different design from that of the government prescribed single pit pour flush subsidised latrines built under the TSC in study villages. Privately financed toilets were complete with full height walls, a roof, and a door or screened entrance to maintain privacy. The toilet cabin was installed on a raised platform, often several feet above ground level, rather than at or close to ground-level as were most of the subsidized off-set pour-flush latrines, so that the latrine could be used in all seasons and throughout the year, even during flooding.

SF latrines we observed were in proper functioning condition with nearby permanent availability of water either in a large water container in the latrine, and/or at a private water source, usually a private shallow tube-well or tap, located very close by or inside the latrine, and these households reported that each capable member, irrespective of gender, fetched their own water for using the latrine and bathing at home afterwards. Several SF and GOI subsidized latrines which had been self-improved had an attached bathroom cubicle (as mentioned above) or had a private place for bathing near the latrine in the back yard, for the convenience of household members to accomplish post-defection bathing and washing purification rituals without having to walk sometimes long distance to public ponds or other open water bodies for these rituals. As explained earlier, the need and importance of sufficient quantities of easily accessible water at the latrine for accomplishing sanitation rituals was one of the primary reasons cited for rejecting the government’s subsidised toilets, and most of the SF and improved GOI subsidized latrines had some kind of private water supply at the house or in the latrine to avoid having to go and fetch water from public sources each time they defecated or entered the latrine.

With an aim to use their latrine for many years, SF latrine owners reported building a large below ground septic tank or two deep pits in series. This was also done so all the family members could use the facility in the morning without the tank or pit filling up with water and backing up into the pan. As a result of their elevated pans and larger pit water-holding capacities, during rising water tables and floods, the below-ground plumbing of SF latrine designs continued functioning without problems in contrast to the subsidized toilet designs in which contaminated water was reported to rise up from the latrine pit into the squat pan blocking use and attracting insects in several facilities in the village of FGD #5, several of which we observed and confirmed.

The need to empty full pits was not reported to be a problem or constraint on latrine usage by household members among SF owners, and several reported having either emptied their pit themselves (including two Brahmin men, FGD #4) after removing the cover and letting the contents dry for several days, or provided drinks or pay (typically Rs 500–600, and up to Rs 2000 plus soap and oil, for a large tank after many years of use) to someone locally who could be called on to empty the dried contents. (FGDs #1, 4, & 8) In contrast, owners of GOI subsidized toilets, whether self-improved or not, often indicated that the pits would need to be emptying frequently (*e.g.*, every year) if all members used the latrine due to the very small size of the pit (see above), however, there was no perceived lack of people locally available to empty pits for a fee. (FGDs #1, 8, & 9)

## Discussion

In this study, we used qualitative methods to explore and develop an in-depth understanding of different factors responsible for low adoption of latrines in rural areas in Odisha, India, notably the lower rates of use of government subsidised latrines implemented by local NGOs under the TSC programme, and preferences for open defecation. We found extensive evidence that even where people had an option to use a household latrine, many were reluctant to adopt latrine use habits and instead chose to continue their traditional behaviours to defecate under the open sky. A clear preference for open defecation in rural areas, particularly by members of households with a GOI subsidized latrine, has also been documented across five northern states of India [22] and confirmed separately for Puri in a study applying the Safe San Index, a new metric to measure person-level latrine use and open defecation rates [23].

We found different reasons for why government subsidised latrines (facilitated by NGOs under TSC) largely remaining unused and rejected. Even villages officially attaining Open Defecation Free (ODF) status were not OD free, as was evident from the practices of participants of FGD #5. These results are consistent with earlier findings of TSC in other parts of India [9, 24–27].

### Gender, age and caste

Men in our study who defecated in the open stated that latrine use did not suite their daily routines, and that latrines were meant for females, as they stay at home most of the time and thus have more need for them. In general, users of latrines were viewed by study participants to be mainly women, especially the newlywed daughter-in-law. There are increasing cases reported of latrine building in rural Odisha, as we observed in the study population, where the prime reason for the latrine installation was the arrival of the newly wed bride in the household. Although there is no evidence of efforts to apply the Community-Led Total Sanitation approach in Puri, as has been undertaken elsewhere in Odisha [28], or of social campaigns like ‘no toilet, no bride’ in Haryana [29], or use of messages around shame, dignity and security of females to promote latrine uptake, male heads of household and future husbands in our study showed more concern for protecting and preserving the dignity, privacy and security of their new daughter-in-law/bride when deciding to install a latrine. They did not want these young women to be seen while they defecated outside because it lowered the prestige of the family. In contrast, similar thinking or motivations were not observed in regard to their daughters or other females within the family. Indian and rural Puri society is still male-dominated, household decisions are taken by men, and females’ needs are rarely attempted to be understood, recognised or addressed by male heads [20]. Thus, policies aiming at empowering women in decision making could be fruitful in enabling females to demand for a life with dignity [30]. Female education and older age at marriage have been found to be key factors associated with greater empowerment of new daughters-in-law in decision-making and agency over their daily lives in their in-laws’ home [31] and thus may be important elements of such policies.

In many of the SF latrine households who tended to be wealthier or better educated upper caste families who had some prior exposure to latrines, daughters-in-law seemed to express gratitude for the ease and convenience of using the latrine (which typically always included a water supply and private place to bath), and for the liberation from worry of being publicly seen bathing as much as open defecating, a situation which could generate village gossip and family shame. On the other hand, married women in GOI subsidized latrine households who tended to be of low and middle castes with little outside exposure, going for open defecation in the evening provided many of them with one of the rare daily opportunities to escape the house, the scrutiny of the mother-in-law, and the confines of their hamlet and socialize with women friends and peers. This was most strongly expressed by married women who were daughters-in-law (*i.e.* not yet mothers-in-law), and a few young ones expressed open regret for having to use the household latrine (FDG #5). This revealing finding is consistent with what some Indian researchers and experts have suggested, that the traditional role of women and rigid code of conduct for them within marriage, can be highly self-limiting, restrictive, and even boring, and contributes directly to the higher observed rates of depression among married women than married men across Indian society [20].

With attainment of mother-in-law status and old age, women were less concerned about being seen open defecating. As roles in the household shift with mother-in-law status, and women gain greater freedom of movement and control over their daily routines compared to daughters-in-law [19], mothers-in-law may be more able to choose where they go and what they do. Other studies of GOI subsidized latrine use have also found that older compared to younger married women in rural India are more likely to defecate in the open [23]. The exception to this pattern among older members was due to disability, immobility, or sickness which made open defecation difficult, similar to observations of reasons for early adoption in Benin [32] and reported elsewhere in India [33].

A study in Tamil Nadu found women and men had different defecation sites [18], and the same was found in our study in rural Puri. Unlike media reports from Northern India, we found little evidence that women saw or experienced going for open defection as a safety problem or threat to their well-being. Social cohesion and fear of reprimand in the study villages appeared strong enough to prevent individual men from molesting women on their way to the open defection sites.

While many studies of latrine use in rural India have observed a stronger tendency for adult women than men to use latrines (*e.g.* [10, 22, 23]), this study has revealed contrasting preferences for open defecation and an unexpectedly complex diversity of views and attitudes towards latrine use held by rural Indian women themselves, sometimes quite negative, which were found to vary with their age, marital status, caste, education, and role/status within the home. These insights suggest a universal preference among females in rural India for using latrines cannot be assumed, and that increased opportunities for social engagement and interaction outside the home for rural women, especially married women of lower socio-economic status, may need to be created so that open defecation no longer serves this purpose if rural women are to fully embrace latrine adoption and use. Others have pointed to the need to increase understanding of the negative health implications of open defecation as important for behaviour change [22, 34]. Separate and concerted efforts focused specifically on how to change social norms of open defecation among rural men, given its greater convenience to them, will also clearly be needed.

### Cultural pollution and purification beliefs and rituals

Although lack of finance and poor quality of government’s subsidised latrines are constraints for not adopting latrines, our results show that primarily old habits and strongly ingrained beliefs around impurity and pollution and the required rituals for purification and cleansing post-defecation in Indian society may play a big part in the choice to continue defecating in the open in the study area. Faeces have always been considered ritually impure as well as physically filthy and water as the necessary medium of purification and ritual cleansing in Indian society [21, 35]. Bathing and clothes changing rituals are deeply ingrained practices post-defecation and after many other kinds of ritual defilement in Indian society [21, 35]. Together these cultural beliefs and practices explain the strong importance households have placed on the need for water provisioning inside the latrine to accomplish required cleansing acts following defecation [27]. Ritual pollution may extend to simply touching or entering the latrine for some higher castes [21, 35], as was described by Brahmin participants in our SF latrine group. This clearly poses a considerable barrier to safe child faeces disposal in the latrine as well as latrine cleaning if elaborate water purification rituals are perceived to be too time-consuming or difficult to perform, added to arguments for providing water availability in the latrine. This possibility is supported by findings from a survey of child faeces disposal practices in rural Indian households with a functioning latrine, that water availability on the premise for using the latrine was associated with safe child faeces disposal [36].

Beliefs that faeces are impure also caused a few participants to consider the practice of containing faeces in the latrine pit in the house as a ‘sin’ , because idols and pictures of gods that are revered are kept and worshipped in every house; having toilets within or next to the house makes the entire house impure. These kinds of strong traditional beliefs can hold back people from adopting the new practice of defecating safely inside latrines [26, 33, 37]. The importance of considering cultural beliefs, however, has long held true for changing sanitation around the globe [38, 39].

### TSC latrine design and implementation

While traditional habits and socio-cultural barriers may be contributing to the present day situation, several studies and reports have drawn attention to serious problems in the TSC programme design and implementation [2]. Inadequate inefficient programme implementation, unprofessional and ad-hoc target-making and inadequate institution building are also some of the reasons contributing to unchanging traditional behaviour [4]. We also found substantial problems with inadequate and inappropriate design and incomplete and sometimes poor quality construction of the TSC subsidized latrines which posed real barriers to latrine use. For example, near-annual risks of monsoon flooding and widespread inundation in the Puri District study area were not considered in the design and construction of the subsidized latrines, many of which had pans installed at or near ground-level and very small, shallow pits compared to SF latrines in the same communities (which typically had elevated pans and large pit volumes). As a result, many of the subsidised toilets were inundated or water-logged, and unusable in the rainy season. In their study across rural north India, Coffey et al. [22] also found that SF latrines had significantly larger pits than GOI subsidized latrines, and that latrines used by all household members were much more likely to have larger pits than those used only by some or few members. A desire for large dry pit volumes has also been observed in Africa, the motivation expressed being to maximize the investment in building the structure and serve the whole family for many years before the pit becomes full and has to be replaced [40, 41].

Others have criticised the single model technology and pointed at the structural deficiencies in the subsidy driven sanitation intervention promoted by the Indian government [3]. Although participants did not mention this explicitly, their non-involvement in shaping the toilet design to suit their needs and preferences may have been a strong reason for discarding their subsidized latrines in our study area. This phenomena was observed elsewhere in rural India in which people who had not been involved in choosing their sanitation technology persisted in their habit of open defecation [3], and has been confirmed in a quantitative study showing individuals in households that had been involved in the choice of their latrine design were 49 % less likely to practice any OD than members of households that had not [23]. The TSC GOI’s individual household latrine unit design of 5 feet wall height, single cubicle, and single shallow pit pour flush latrine with no roof and no water provision and, in many cases, with doors missing [2], was regarded by people as incomplete and insufficient for use. Among the study population of rural Odisha, however, we found people not using a GOI subsidized latrine even if complete (as per government guidelines) and functional, but lacking a roof. Owners expected to receive more subsidies sometime later, so delayed using the facility, or completing the facility at their own costs. The long history of experience with hardware subsidies in sanitation programming has shown that toilet construction subsidies do not guarantee that toilets will be used and are a poor substitute for creating real demand [42]. As per TSC guidelines, the subsidy was meant as an incentive for backward families, which was to be reimbursed only after the completion of the toilets. High reliance on the subsidies however has been observed among rural Indian families [2, 3, 30], and the subsidy amount reported as inadequate to construct an acceptable functional sanitation system [43]. In contrast, there is evidence of poorer households in India achieving higher levels of sanitation on their own [44].

Lack of provision for any water supply in the units emerged as a major factor for non-use in the design of facilities in the study setting, given the quantities of water needed for anal cleansing, flushing and sanitation purification rituals. Participants were optimistic that usage would increase among existing GOI subsidized latrine households with provision of water in the latrines. Our findings corroborate those of other Indian studies in Rajasthan [30], and Tamil Nadu [26, 45] which found that absence of water at the latrine for post-defecation anal cleansing and bathing (which is crucial to accomplish customary sanitation purification rituals described above) reduced latrine uptake and use. In places where the distance of water supply points was more than 500 metres from the latrine, villagers have shown unwillingness to fetch water [46]. In rural Madhya Pradesh, lack of a water connection was the second most frequent reason (excluding lack of money) for not having a toilet facility [47]. In a study using the Safe San Index to measure consistent latrine use in Puri District, a water source in the latrine was associated with a 2 fold increase in safe excreta disposal rates (*i.e.*, defecation and disposal in the latrine) across all members, compared to latrine owners with a public water source located outside the compound [23]. Water requirements for cleansing and purifying rituals mean that unavailability of water supply in sanitation facilities will continue to be a major shortcoming of the subsidised latrines, unless addressed. O’Reilly et al. [34], in taking a politically ecology approach to understanding sanitation adoption in rural Indian, has argued for the critical importance of inaccessibility of water as an important ecological and structural constraint to be addressed. A global review of determinants of rural latrine use and open defecation behaviour has also highlighted the importance of accessible and reliable water availability as a factor in latrine adoption [48]. People will continue to do what was convenient and easy, and open defecate near local surface water bodies (ponds and rivers) [49].

We found many interesting patterns of continued open defecation among different groups even among households with access to latrines, including self-financed owners, establishing the fact that only a small percentage of people in rural areas seemed motivated to build and use latrines. It may be less about sanitation unawareness and more about the benefits and drivers of continued open defecation that have failed to bring about a shift in thinking about safe disposal of faeces. Lack of awareness on the health adversities caused by unsafe faeces disposal is also a pressing challenge in rural India [6]. The habit of defecating in an open environment without walls [49], lack of privacy provided by poorly designed and incomplete GOI toilets, absence of water in the latrines, purity and sanitation rituals, extra work and effort associated with latrine use, and socialising especially for married women while going for defecation, are some of the strong drivers of continued defecation in fields and open areas rather than in a household latrine, despite access.

A shift in thinking about sanitation and latrines, and a change in old sanitary habits are possible if people are taken through the experiences of the negative impacts of open defecation. Efforts and the approaches to motivate these changes in thinking have not generally been rigorously undertaken by the TSC program, such that the direct link between using a sanitation facility and its benefits remained unclear to most open defecators [38]. If they had made these connections, participants would not have developed the perception that walking long distances to the defecation sites is good for health or that defecation outside is not unhealthy.

There were segments of the rural population who were found to be regular users. These people tended to be more educated, informed, had a higher financial status, travelled more and had greater awareness on the benefits of using latrines. They generally belonged to higher castes who traditionally have better financial status, better access to formal education, and are more likely to obtain jobs in towns and cities that expose them to a different living style, including to latrines, as has been observed with early latrine adopters elsewhere [32]. For these reasons, in the study population most toilet adopters were found to have lived in cities or served in government jobs where they had opportunities to become acquainted with using latrines. Realising the importance of past exposure in latrine adoption, future programmes may attempt extensively to work on the exposure aspect of toilet promotion. This was suggested by one of the participants who served as a teacher in different parts of the state.

While lack of sanitation may seem to be a basic problem, with a seemingly easy solution, in reality it is far more complex to implement successfully than would seem at first. There are underlying factors like beliefs, old habits, and rituals that complicate the success of sanitation interventions and impact toilet uptake [38]. Extensive research to understand the relationship between dynamics of individuals and societal dynamics with regard to defecation and new sanitation behaviours are needed before implementation. These findings may help in development of sustainable strategies for motivating people to build and use toilets in rural Odisha and other places in rural India where traditional open defecation is entrenched.

## Conclusions

Our findings suggest that absence of latrine infrastructure is not a primary factor for continued open defecation and that toilet building alone will not address the widespread problem of open defecation in rural India. Poor quality and an inappropriate and single latrine design made available to rural people under government sanitation schemes may be important factors but are not the sole reason for low latrine uptake and use. There are other behavioural aspects which constrain the adoption and use of latrines. These behavioural aspects vary with communities, across gender and different age groups and castes. Any future sanitation intervention, instead of achieving targets, needs to consider these aspects and approach the issue of sanitation behaviour change holistically.

## Abbreviations

CRSP: Central Rural Sanitation Programme
GOI: Government of India
NBA: Nirmal Bharat Abhiyan
NGO: Non-governmental Organisation
OD: Open Defecation
RATS: Relevance of study question appropriateness of qualitative method, transparency of procedures, soundness of interpretive approach
SF: Self-financed
TSC: Total Sanitation Campaign
